# Immunogenic Cell Death Induced by EEO in Breast Cancer Cells Promotes Dendritic Cell Activation in a CRT-Dependent Manner

**DOI:** 10.3390/life16091466

**Published:** 2026-09-02

**Authors:** Seong-Ah Shin, Sun Young Moon, Seyeon Choi, Minji Kim, Moonsu Kim, Jun Hyuck Lee, Seulah Lee, Ki Hyun Kim, Hyun Ho Park, Ui Joung Youn, Chang Sup Lee

**Affiliations:** 1 College of Pharmacy and Research Institute of Pharmaceutical Sciences, Gyeongsang National University, Jinju 52828, Republic of Korea; shinsaya@gnu.ac.kr (S.-A.S.); symoon0414@gnu.ac.kr (S.Y.M.); seyeon0608@gnu.ac.kr (S.C.); minji8038@gnu.ac.kr (M.K.); kk10022@gnu.ac.kr (M.K.); 2 Department of Polar Sciences, University of Science and Technology, Incheon 21990, Republic of Korea; junhyucklee@kopri.re.kr (J.H.L.); ujyoun@kopri.re.kr (U.J.Y.); 3 Research Unit of Cryogenic Novel Material, Korea Polar Research Institute, Incheon 21990, Republic of Korea; 4 Department of Convergent Biotechnology and Advanced Materials Science, BK21 Interdisciplinary Program in IT-Bio Convergence System, Kyung Hee University, Yongin 17104, Republic of Korea; lee.seulah@khu.ac.kr; 5 School of Pharmacy, Sungkyunkwan University, Suwon 440-746, Republic of Korea; khkim83@skku.edu; 6 College of Pharmacy, Chung Ang University, Seoul 06974, Republic of Korea; xrayleox@cau.ac.kr; 7 Division of Life Sciences, Korea Polar Research Institute, Incheon 21990, Republic of Korea

**Keywords:** (3β,5α,8α)-5,8-epidioxyergost-6-en-3β-ol, breast cancer, immunogenic cell death, calreticulin, dendritic cell, cold tumor, phagocytosis, maturation

## Abstract

Cancer immunotherapy faces significant challenges in treating “cold” tumors, which respond poorly to current strategies. Breast cancer is a major cause of cancer-related mortality in women and is a representative immunosuppressive cold tumor. The luminal A subtype of breast cancer exhibits an immune-cold phenotype, with MCF-7 cells used as a representative cell model. Here, we identified (3β,5α,8α)-5,8-epidioxyergost-6-en-3β-ol (EEO), isolated from *Gymnopilus orientispectabilis*, as an inducer of immunogenic cell death (ICD) hallmarks in MCF-7 cells, including cell surface exposure of calreticulin (CRT) and the extracellular release of damage-associated molecular patterns (DAMPs), such as ATP and High Mobility Group Box 1 (HMGB1). These ICD-associated events subsequently promoted CRT-dependent dendritic cell (DC) activation in a co-culture system with bone marrow-derived DCs and MCF-7 cells. Furthermore, surface-exposed CRT enhanced the phagocytosis of EEO-treated MCF-7 cells by DCs, and this phagocytic activity promoted DC maturation, as indicated by increased cell surface levels of the maturation markers major histocompatibility complex class II (MHC II) and cluster of differentiation 86 (CD86). Moreover, CRT knockdown in MCF-7 cells reduced surface CRT exposure following EEO treatment, thereby suppressing DC-mediated phagocytosis and subsequent DC maturation. Taken together, these findings suggest that EEO is a promising candidate for enhancing the antitumor efficacy of existing breast cancer immunotherapies through ICD-mediated DC maturation and converting immunologically cold tumors into hot tumors.

## 1. Introduction

Breast cancer is the most frequently diagnosed malignancy in women worldwide and remains a leading cause of cancer-related deaths [1]. Although conventional therapies for breast cancer have improved, the disease still continues to pose a major public health challenge [2]. Recently, immunotherapy, which harnesses the immune system, has emerged as a promising strategy for treating malignancies [3]. However, the clinical efficacy of immunotherapy remains limited in a substantial proportion of patients with solid tumors, including breast and gastric cancers, owing to tumor heterogeneity and the immunosuppressive tumor microenvironment (TME) [4]. Moreover, breast cancer is generally considered an immunologically “cold” tumor, characterized by limited infiltration and activation of cytotoxic T lymphocytes (CTLs) within the TME, thereby reducing the therapeutic response to immunotherapy [3,4]. The insufficient recruitment and activation of immune effector cells within the TME further contribute to immune evasion [5,6]. Therefore, converting immunosuppressive “cold” tumors into immune-activated “hot” tumors represents a promising strategy for improving the efficacy of breast cancer immunotherapy.

One strategy to achieve this conversion is the induction of immunogenic cell death (ICD) [7,8]. ICD is a regulated form of cell death that triggers an adaptive immune response in response to specific stimuli and can be triggered by certain chemotherapeutic agents, including anthracyclines (e.g., doxorubicin, daunorubicin, and mitoxantrone) and oxaliplatin [9,10,11]. Mechanistically, cells undergoing ICD expose calreticulin (CRT) on their surface and release immunostimulatory damage-associated molecular patterns (DAMPs) along with cytokines, which are recognized as the hallmarks of ICD [8,12]. CRT, a multifunctional chaperone protein, is primarily found within the lumen of the endoplasmic reticulum (ER) under normal physiological conditions [7]. Upon translocation to the cell surface, CRT functions as an “eat-me” signal that promotes the phagocytic uptake of dying cells by dendritic cells (DCs), ultimately enhancing cross-presentation of tumor antigen and priming of tumor-specific CTLs [13]. In particular, CRT expression has been reported to be positively associated with CTL and DC infiltration in various types of solid cancers, including breast, pancreatic, and colon cancers [14,15]. Extracellular ATP, one of the DAMPs released during ICD, functions as a “find-me” signal by recruiting innate immune cells, including DCs and macrophages, through binding to P2Y2 receptors [9]. Another DAMP, High Mobility Group Box 1 (HMGB1), exerts potent proinflammatory effects by interacting with the cognate receptor Toll-like receptor 4 (TLR4) on DCs, thereby promoting the efficient processing and cross-presentation of tumor antigens from dying cells [16]. As our understanding of ICD in cancer continues to expand, ICD-driven therapeutic strategies are increasingly recognized as promising next-generation approaches for enhancing cancer immunotherapy.

**Figure 1:**
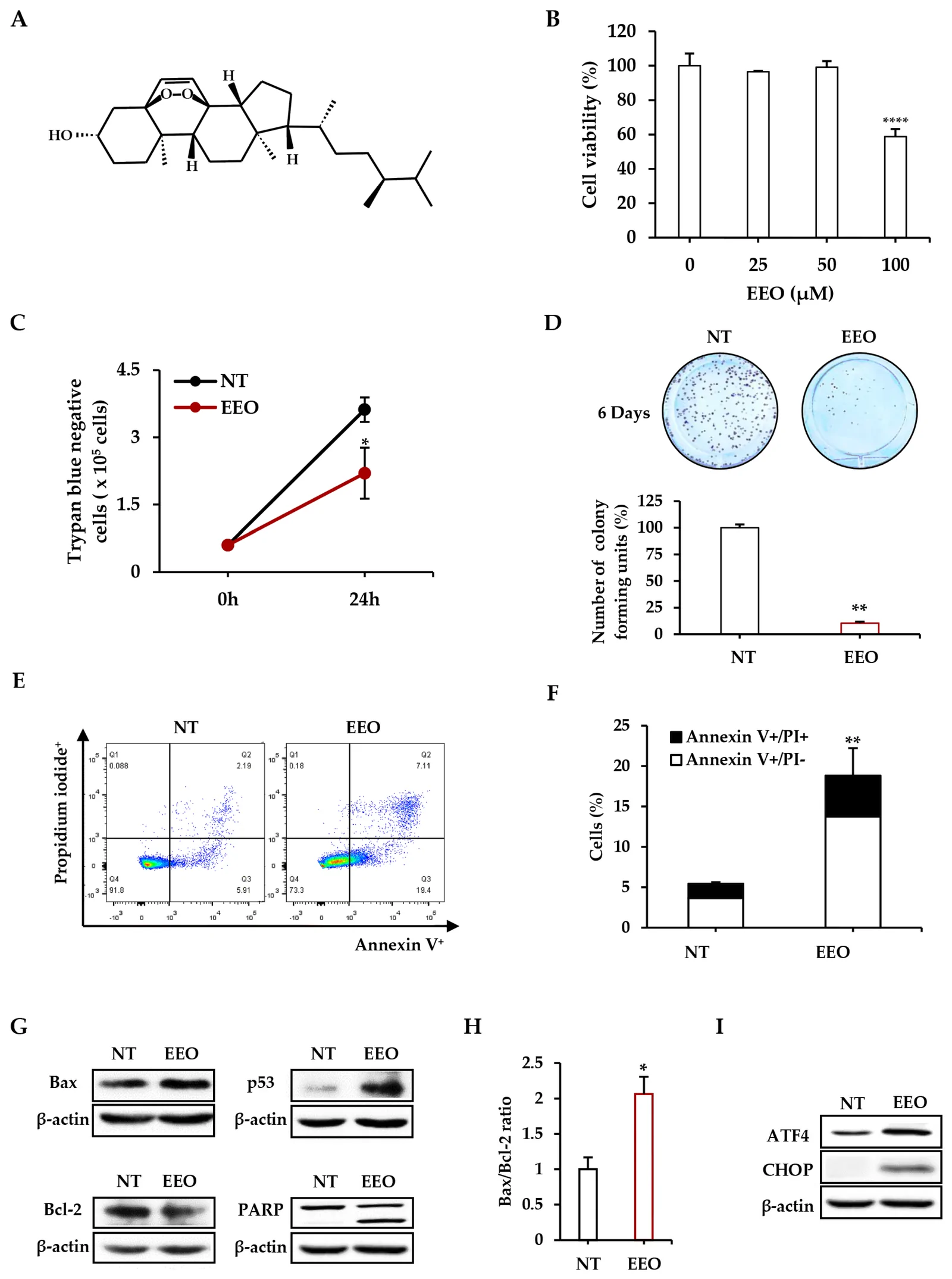
EEO inhibits cell viability and triggers apoptosis in MCF-7 breast cancer cells. (**A**) chemical structure of (3β,5α,8α)-5,8-epidioxyergost-6-en-3β-ol (EEO). (**B**) MCF-7 cells were treated with EEO (25, 50, or 100 µM) for 24 h, and cell viability was assessed using the MTT assay. (**C**) Following treatment with EEO for 24 h, viable cells were counted by trypan blue staining using a hemocytometer. (**D**) MCF-7 cells were treated with EEO, maintained in culture for 6 d, and subsequently stained with crystal violet to visualize colony formation. Colony numbers were quantified using ImageJ software (v1.50i). (**E**,**F**) Following EEO treatment for 24 h, cells were stained with Annexin V-FITC/propidium iodide (PI), and apoptosis was analyzed by flow cytometry. The percentage of apoptotic cells was quantified from the flow cytometric data. (**G**–**I**) MCF-7 cells were treated with or without EEO for 24 h. (**G**) The expression level of apoptosis-related proteins (p53, Bax, Bcl-2, and cleaved PARP) was analyzed by Western blot. (**H**) Quantification of Western blot band densitometry showing the Bax/Bcl-2 ratio. (**I**) The expression levels of ER stress-related proteins (ATF4 and CHOP) were analyzed by Western blot. Data are presented as the mean ± standard deviation (SD). Statistical significance was assessed by one-way ANOVA followed by Dunnett’s multiple comparisons test against the untreated control (**B**; *F*(3,8) = 57.76, *p* < 0.0001), or by unpaired two-tailed Student’s *t*-test (**C**, *t*(4) = 3.888, *p* = 0.0177; **D**, *t*(2) = 36.25, *p* = 0.0008; **F**, *t*(4) = 5.959, *p* = 0.0040; **H**, *t*(6) = 3.506, *p* = 0.0127). * *p* < 0.05, ** *p* < 0.01, and **** *p* < 0.0001 versus the untreated group.

Accumulating evidence indicates that numerous natural compounds possess significant potential for cancer immunotherapy by remodeling the immunosuppressive TME [17,18]. Furthermore, natural compounds have attracted considerable attention as promising inducers of ICD in cancer therapy and represent a promising avenue for the development of novel ICD-based immunotherapies [19]. However, only a limited number of natural compounds have been identified as ICD inducers, and the discovery of novel ICD-inducing compounds continues to pose a major challenge.

(3β,5α,8α)-5,8-epidioxyergost-6-en-3β-ol (EEO) (Figure 1A) is a natural sterol isolated from *Gymnopilus orientispectabilis* [20]. EEO has recently been reported to exhibit antimicrobial activity [20]; however, its potential role in ICD-mediated antitumor immunity remains unknown. Our findings indicate that EEO induces ICD, accompanied by apoptotic features, and promotes DC activation through the phagocytosis of ICD-induced breast cancer cells, suggesting its potential as an immunomodulatory strategy for treating immunologically “cold” tumors.

## 2. Materials and Methods

### 2.1. Reagents

EEO was provided by the Korean Polar Research Institute (KOPRI). EEO is a single compound isolated from the fruiting bodies of *Gymnopilus orientispectabilis*, with a purity of >95% as determined by ^1^H-NMR spectroscopy [20]. EEO was dissolved in dimethyl sulfoxide (DMSO). Fetal bovine serum (FBS) and Roswell Park Memorial Institute (RPMI) 1640 medium were obtained from Corning (Corning, NY, USA), and penicillin–streptomycin–glutamine (PSQ) was purchased from Gibco (Grand Island, NY, USA). DMSO and thiazolyl blue tetrazolium bromide (MTT) were obtained from Sigma-Aldrich (St. Louis, MO, USA). All primary and secondary antibodies used in this study were obtained from Cell Signaling Technology (Danvers, MA, USA), BioLegend (San Diego, CA, USA), BD Pharmingen (San Diego, CA, USA), Invitrogen (Waltham, MA, USA), and Seracare (Gaithersburg, MD, USA).

### 2.2. Cell Culture

The human breast cancer cell line MCF-7 was provided by Gongju National University (Gongju, Republic of Korea). The cells were routinely maintained at 37 °C in a humidified 5% CO_2_ atmosphere, in RPMI 1640 medium containing 10% FBS and 1% PSQ.

### 2.3. Small Interfering RNA (siRNA)-Mediated Knockdown

siRNA targeting CRT was purchased from Bioneer (Daejeon, Republic of Korea). Cells were cultured to approximately 60% confluence, and transfected with siRNA at a final concentration of 30 nM via Lipofectamine 3000 (Invitrogen) reagent, following the manufacturer’s instructions. Cells transfected with a non-targeting siRNA (Bioneer, SN-1002) served as the negative control. CRT knockdown cells were subsequently confirmed by RT-PCR and Western blot analysis, as described below.

### 2.4. Cell Viability Assay

Cytotoxicity was evaluated by MTT assay. MCF-7 cells were seeded at a density of 2 × 10^4^ cells/well in 48-well plates and incubated for 24 h. The cells were then treated with EEO at concentrations of 25, 50, and 100 µM for 24 h. MTT solution (1 mg/mL) was then added to each well and incubated for 2 h at 37 °C, after which the formazan crystals were solubilized in 200 µL DMSO. Absorbance was measured at 570 nm using a microplate reader (Varioskan LUX, Thermo Fisher Scientific, Waltham, MA, USA).

To further evaluate the effect of EEO on cell number, trypan blue staining was performed. MCF-7 cells were seeded in 12-well plates at a density of 0.6 x 10^4^ cells/well and treated with EEO for 24 h. The cells were harvested by trypsinization and stained with trypan blue. Viable cells (trypan blue-negative) were counted using a hemocytometer.

To assess the effects of EEO on cell viability and proliferative capacity, a colony formation assay was performed. MCF-7 cells were seeded in 6-well plates at a density of 1.5 × 10^5^ cells/well and treated with EEO (100 µM) for 24 h. The EEO-containing medium was then replaced with fresh medium, and cultures were maintained for an additional 6 days. The cells were subsequently fixed with 4% paraformaldehyde (PFA) for 10 min and stained with 0.05% crystal violet for 5 min. Following washing, colony images were captured and quantified using ImageJ software (v1.50i).

### 2.5. Apoptosis Detection Analysis

MCF-7 cells were seeded at a density of 5 × 10^4^ cells/well in 12-well plates and cultured for 24 h before treatment with EEO (100 µM) for an additional 24 h. For CRT knockdown experiments, cells were treated with EEO for 24 h post-transfection with either CRT-targeting or negative control siRNA. Following treatment, the cells were collected and labeled with FITC-Annexin V together with propidium iodide (PI), incubating for 15 min at room temperature (RT). Apoptotic cells were detected by flow cytometry, and Flow cytometry was used to quantify the proportion of apoptotic cells, with data analyzed in FlowJo (v10).

### 2.6. Western Blot

To analyze protein expression, whole-cell lysates were prepared in radioimmunoprecipitation assay (RIPA) buffer supplemented with protease and phosphatase inhibitors. After sonication, insoluble material was removed by centrifugation at 13,000× *g* for 10 min at 4 °C. Total protein in each supernatant was quantified via the Bradford method, and 25 µg per sample was resolved by 12% SDS-PAGE prior to transfer onto nitrocellulose membranes. Non-specific binding was blocked with 5% skim milk (30 min), after which blots were incubated overnight at RT with one of the following primary antibodies: anti-β-actin, anti-p53, anti-B-cell lymphoma 2 (Bcl-2), anti-BCL2-associated X protein (Bax), anti-poly (ADP-ribose) polymerase (PARP), anti-activating transcription factor 4 (ATF4), and anti-C/EBP homologous protein (CHOP). β-actin served as the loading control. After washing, a horseradish peroxidase-conjugated secondary antibody was applied for 1 h at RT. Target proteins were detected using enhanced chemiluminescence reagents and visualized using an iBright™ CL1500 Imaging System (Applied Biosystems, Waltham, MA, USA).

### 2.7. Determination of Cell Surface CRT Exposure

Cell surface exposure of CRT was assessed by flow cytometry and confocal laser-scanning microscopy (CLSM)(Olympus, Tokyo, Japan). MCF-7 cells were plated at a density of 5 × 10^4^ cells/well in 12-well plates and cultured for 24 h prior to treatment with EEO (100 µM) for 24 h. To examine the contribution of CRT, cells were transfected with CRT-targeting or negative control siRNA 24 h prior to EEO treatment. For flow cytometric analysis, cells were fixed with 0.25% PFA for 5 min at RT, blocked for 30 min, and incubated with an Alexa Fluor 488-conjugated anti-CRT antibody at 4 °C in the dark for 1 h. PI was then added to exclude membrane-permeabilized cells. Cell surface CRT expression was assessed by flow cytometry using FlowJo software. For CLSM, cells were seeded on coverslips in 12-well plates and similarly treated with EEO for 24 h. Following fixation with 4% PFA for 10 min, the cells were washed with cold PBS. After blocking for 30 min, the cells were labeled with the Alexa Fluor 488-conjugated rabbit anti-CRT antibody for 2 h in the dark and washed with 2% bovine serum albumin (BSA). The coverslips were then mounted onto slides, and fluorescence images were acquired using CLSM.

### 2.8. RNA Isolation and qRT-PCR Analysis

Total RNA was purified with TRIzol reagent (Invitrogen, Carlsbad, CA, USA), and the RNA concentration and purity were determined using a NanoDrop spectrophotometer (Thermo Fisher Scientific). For cDNA synthesis, 2 µg of total RNA was used with the cDNA Synthesis Platinum Master Mix (GenDEPOT, Katy, TX, USA) following the manufacturer’s instructions. qRT-PCR was performed using the StepOnePlus real-time PCR system (Applied Biosystems, Foster City, CA, USA) with Power SYBR Green Master Mix (Applied Biosystems), and the gene-specific primers listed in Table 1. The threshold cycle (Ct) values were normalized against β-actin, and relative gene expression levels were calculated using the 2^−ΔΔCt^ method.

### 2.9. DAMP Release Analysis

To assess intracellular ATP levels following EEO treatment (100 µM), MCF-7 cells were plated in 24-well plates and treated with EEO for 6 h. Cells were then treated with the ATP-sensitive fluorochrome quinacrine (15 µM) containing Hoechst 33342 (1 µg/mL) for 30 min at 37 °C. Green fluorescence signals were visualized under a fluorescence microscope (400×) and analyzed using ImageJ software.

To evaluate HMGB1 release, MCF-7 cells were cultured on poly-L-lysine-coated coverslips in 6-well plates and treated with or without EEO (100 µM) for 24 h. The cells were then fixed with 4% PFA for 10 min and rinsed with 2% BSA. The cells were bound with anti-HMGB1 antibody for 2 h at RT, washed with 2% BSA, and subsequently incubated with an Alexa Fluor 555-conjugated goat anti-rabbit IgG for 1 h at RT in the dark. The nuclei were visualized using Hoechst33342 for 5 min and mounted onto slides. Fluorescence images were captured using a fluorescence microscope (×400) and analyzed using ImageJ software.

### 2.10. Isolation of Mouse Bone Marrow (BM) Cells and Generation of BM-Derived DCs (BMDCs)

BM cells were isolated from C57BL/6 mice (*n* = 6 (3 for Figure 3 and 3 for Figure 4), 8–9 weeks old (Koatech, Pyeongtaek, Republic of Korea)) for co-culture experiments with MCF-7 cells, with one mouse per independent experiment. BM cells were not pooled; each biological replicate was independently prepared from an individual mouse. BM cells were isolated from the femur and tibia of each mouse, as previously described [21]. Red blood cells were removed by treatment with RBC lysis buffer for 5 min at RT. The remaining cells were then plated in 6-well plates and maintained in culture medium containing 20 ng/mL granulocyte-macrophage colony-stimulating factor (GM-CSF) and interleukin (IL)-4. Fresh culture medium containing the cytokines was added every 3 d. After 9 days of differentiation, non-adherent and loosely adherent cells were harvested for subsequent analyses. The Institutional Animal Care and Use Committee of Gyeongsang National University approved all experimental procedures used in this study (GNU-240906-M0179).

### 2.11. Phagocytosis Assay

MCF-7 cells, with or without EEO treatment, were labeled with CypHer 5E (1 µM) for 30 min and co-cultured with BMDCs using an effector-to-target cell ratio of 1:10 for 1 h. As a control, untreated MCF-7 cells were incubated together with BMDCs under the identical conditions. To perform CRT knockdown experiments, cells were transfected using CRT-targeting or negative control siRNA 24 h prior to EEO treatment, followed by the phagocytosis assay. Following co-culture, all cells were harvested and treated with anti-mouse CD16/32 antibody for 20 min to block Fc receptors, followed by incubation with FITC-labeled anti-mouse CD11c antibody for 30 min. CD11c^+^CypHer^+^ cells were identified as BMDCs that had engulfed dead MCF-7 cells. Phagocytic activity was quantified by flow cytometry using FlowJo software.

### 2.12. BMDC Maturation Analysis

Following treatment of MCF-7 cells with EEO (100 µM) for 24 h, cells were co-cultured using BMDCs at a 1:5 ratio for an additional 12 h. For CRT knockdown experiments, cells were transfected with CRT-targeting siRNA or negative control siRNA 24 h prior to EEO treatment. Following co-culture, cells were treated with anti-CD16/32 antibody for 20 min to block Fc receptors, followed by staining with FITC-conjugated anti-CD11c, PE-labeled anti-CD86, and APC-labeled anti-major histocompatibility complex class II (MHC II) for 30 min. The expression of DC maturation markers was analyzed by flow cytometry using FlowJo software.

### 2.13. Statistical Analysis

Data are presented as mean ± standard deviation from two or three independent experiments. Prior to one-way ANOVA, normality of the data was assessed using the Shapiro–Wilk test, and all groups satisfied the normality assumption (*p* > 0.05). Differences between each treatment group and the control group were evaluated by one-way analysis of variance (ANOVA) followed by Dunnett’s multiple comparisons test, whereas differences between two groups were evaluated using an unpaired, two-tailed Student’s *t*-test. Statistical significance was defined as * *p* < 0.05, ** *p* < 0.01, *** *p* < 0.001, **** *p* < 0.0001.

## 3. Results

### 3.1. EEO Induces Cytotoxicity Through Apoptosis in MCF-7 Breast Cancer Cells

To date, most agents that induce ICD have also been shown to activate various forms of regulated cell death, such as apoptosis, ferroptosis, necroptosis, and pyroptosis [16]. Accordingly, the cytotoxic effects of EEO were evaluated in MCF-7 breast cancer cells, in order to evaluate its potential as an ICD inducer. The MTT assay demonstrated that EEO reduced the viability of MCF-7 cells at 100 µM, whereas no cytotoxic effects were observed at lower concentrations (Figure 1B). Consistent with these findings, trypan blue staining showed a significant reduction in the number of viable MCF-7 cells following treatment with EEO (100 µM) (Figure 1C). In addition, a colony formation assay was conducted to evaluate the long-term effects of EEO on cell survival. EEO suppressed colony formation, further confirming its cytotoxic activity (Figure 1D). To confirm whether the EEO-induced reduction in cell viability was linked to apoptotic cell death, Annexin V-FITC/PI double staining was performed, followed by flow cytometric analysis. As shown in Figure 1E,F, EEO treatment significantly increased the proportion of apoptotic cells compared with that in the control group. Furthermore, apoptosis-related protein expression levels were assessed by Western blot analysis. EEO treatment significantly increased the expression of the pro-apoptotic proteins p53 and Bax, while decreasing the expression of the anti-apoptotic protein Bcl-2. In addition, EEO promoted the cleavage of PARP, a well-established marker of apoptosis (Figure 1G). Consistent with these findings, the Bax/Bcl-2 ratio was significantly increased in EEO-treated MCF-7 cells compared to untreated controls (Figure 1H). Moreover, EEO treatment increased the expression of the ER stress markers ATF4 and CHOP, suggesting that EEO-induced apoptosis is associated with activation of the ER stress pathway (Figure 1I). Collectively, these findings indicate that EEO suppresses MCF-7 breast cancer cell viability through the induction of apoptosis.

### 3.2. EEO Induces Hallmarks of ICD in MCF-7 Breast Cancer Cells

**Figure 2:**
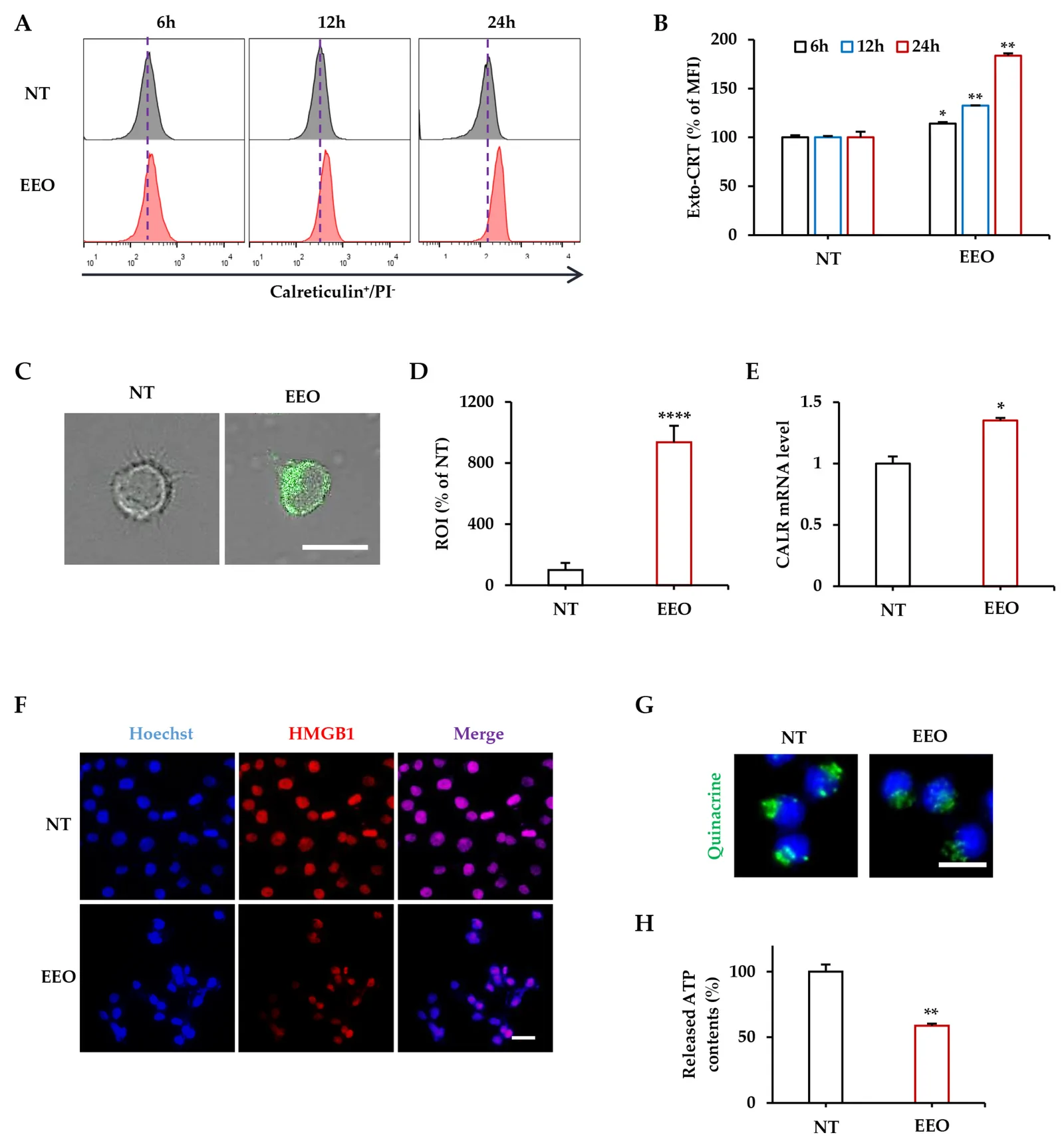
EEO induces cell surface calreticulin (CRT) exposure as well as release of damage-associated molecular patterns (DAMPs) in MCF-7 breast cancer cells. (**A**,**B**) MCF-7 cells were cultured with EEO for 6, 12, or 24 h and stained with FITC-labeled anti-CRT antibody for 30 min. Cell surface CRT expression (relative to NT) was analyzed by flow cytometry using FlowJo software. (**C**) Cell surface-exposed CRT was visualized by confocal laser-scanning microscopy after treatment with EEO for 24 h. Representative images are shown. (**D**) Fluorescence intensity (green) of CRT expression in the region of interest was quantified using Image J software. (**E**) CRT mRNA expression was analyzed by qRT-PCR. (**F**) Intracellular High Mobility Group Box 1 (HMGB1) localization was evaluated by immunofluorescence after 24 h of EEO treatment. Representative images are shown. (**G**,**H**) Intracellular ATP levels were assessed by quinacrine staining (green) 6 h after EEO treatment. ATP-associated fluorescence intensity was quantified in the region of interest using ImageJ software. Representative images are shown. Scale bars = 20 µm. Data are presented as the mean ± SD. * *p* < 0.05, ** *p* < 0.01, **** *p* < 0.0001 compared with the non-treated group.

ICD triggered by chemotherapeutic agents, such as doxorubicin and mitoxantrone, is characterized by several hallmark events, including surface-exposed CRT (translocation of CRT to the cell membrane), as well as the secretion of DAMPs such as HMGB1 and ATP [22,23]. To determine whether EEO induces these ICD hallmarks in MCF-7 cells, cell surface exposure of CRT was examined by flow cytometry. As shown in Figure 2A,B, EEO treatment increased cell surface CRT exposure in a time-dependent manner, reaching approximately a 1.8-fold increase relative to the control group after 24 h. To visualize CRT on the cell surface, immunofluorescence staining followed by CLSM was conducted following 24 h of EEO treatment. EEO-treated cells exhibited markedly stronger CRT fluorescence (green) on the cell surface than untreated control cells (Figure 2C). Quantitative analysis of fluorescence intensity based on the region of interest showed a significant approximately nine-fold increase relative to the control group (Figure 2D). Moreover, a previous study reported increased CRT mRNA levels following treatment with an ICD inducer [24]. Consistent with this, CRT mRNA expression was increased in EEO-treated MCF-7 cells (Figure 2E). Furthermore, HMGB1 and ATP levels were examined by fluorescence microscopy. Immunofluorescence analysis showed a reduction in intracellular HMGB1 fluorescence (red) following EEO treatment (Figure 2F). Similarly, intracellular quinacrine fluorescence (green), which reflects ATP levels, was decreased in EEO-treated MCF-7 cells (Figure 2G), and quantitative analysis verified a marked reduction in ATP fluorescence intensity (Figure 2H). Taken together, these findings suggest that EEO induces key hallmarks of ICD, including surface exposure of CRT along with the secretion of DAMPs (HMGB1 and ATP) in MCF-7 breast cancer cells.

### 3.3. EEO-Treated MCF-7 Breast Cancer Cells Promote Phagocytosis and Maturation of BMDCs

**Figure 3:**
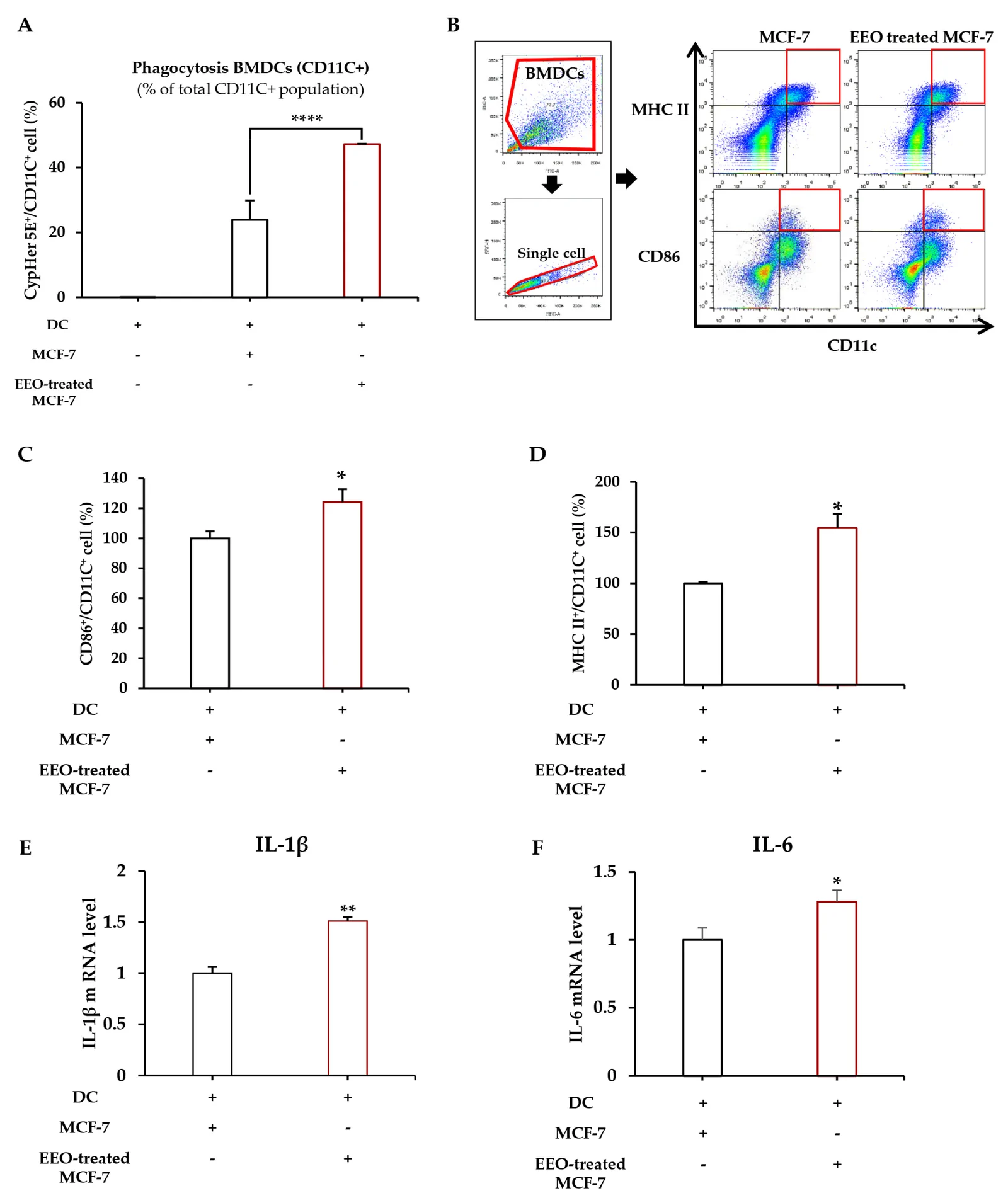
EEO-treated MCF-7 breast cancer cells promote phagocytosis as well as maturation of bone marrow-derived dendritic cells (BMDCs). (**A**) EEO-treated MCF-7 cells labeled with CypHer 5E were co-cultured with BMDCs for 1 h. BMDCs were stained with an FITC-labeled CD11c antibody, and phagocytosis was analyzed by flow cytometry. (**B**) BMDCs were co-cultured with EEO-treated cells for 24 h and stained for DC maturation markers (PE-CD86 and APC-MHC II) and analyzed by flow cytometry. The gating strategy for flow cytometric analysis was as follows: cells were sequentially gated on forward and side scatter (FSC-A/SSC-A) to exclude debris, and on SSC-A/SSC-H to select single cells. Surface expression of (**C**) CD86 and (**D**) MHC II was quantified. (**E**) IL-1β and (**F**) IL-6 mRNA levels were determined by qRT-PCR in mature BMDCs. Data are presented as the mean ± standard deviation (SD). Statistical significance was assessed by one-way ANOVA followed by Dunnett’s multiple comparisons test against the untreated MCF-7 control (**A**; *F*(2,6) = 353.2, *p* < 0.0001), or by unpaired two-tailed Student’s *t*-test (**C**, *t*(4) = 4.404, *p* = 0.0117; **D**, *t*(4) = 3.294, *p* = 0.0301; **E**, *t*(2) = 11.68, *p* = 0.0072; **F**, *t*(4) = 3.150, *p* = 0.0345). * *p* < 0.05, ** *p* < 0.01, and **** *p* < 0.0001 versus the untreated MCF-7 group.

During ICD, DCs, which are key innate immune cells, engulf dying cells by recognizing CRT, a well-established eat-me signal [25]. To investigate whether EEO-induced ICD enhances phagocytosis by DCs, mouse BM cells were isolated and differentiated into BMDCs in culture medium supplemented with GM-CSF and IL-4. BMDCs exhibited greater phagocytosis of EEO-treated MCF-7 cells compared with those co-cultured with untreated MCF-7 cells (Figure 3A). Moreover, DCs that recognize and engulf CRT-exposed dying cells are known to undergo maturation, a process that promotes antigen presentation and contributes to the establishment of a proinflammatory TME [26,27]. As shown in Figure 3B–D, BMDCs co-cultured with EEO-treated MCF-7 cells exhibited elevated expression of maturation markers. Furthermore, IL-1β and IL-6 mRNA expression levels, pro-inflammatory cytokines known to be upregulated during DC maturation, were significantly increased in BMDCs co-cultured with EEO-treated MCF-7 cells compared with those co-cultured with untreated MCF-7 cells (Figure 3E,F). Collectively, these findings suggest that EEO-induced ICD enhances the immunogenic potential of breast cancer cells by promoting BMDC phagocytosis and maturation, consequently contributing to a proinflammatory TME.

### 3.4. EEO-Induced Cell Surface CRT Exposure Is Required for BMDC Phagocytosis and Maturation

**Figure 4:**
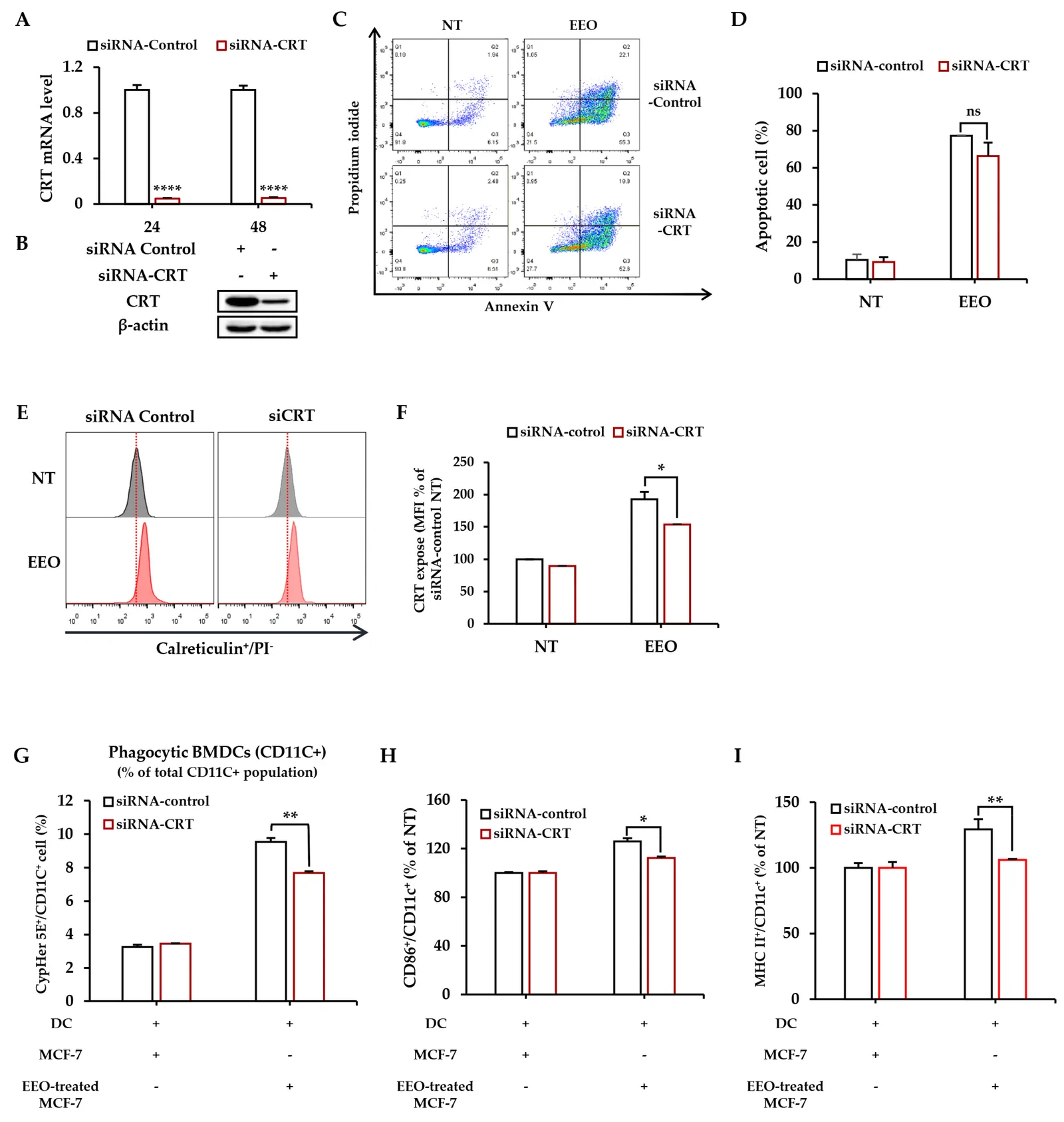
Surface-exposed CRT is required for BMDC phagocytosis as well as maturation. MCF-7 cells were subjected to transfection with small interfering RNA (siRNA) directed against CRT or with non-targeting control siRNA for 24 h. (**A**) CRT mRNA and (**B**) protein expression levels were determined by qRT-PCR and Western blotting, respectively. CRT mRNA expression was evaluated up to 48 h to confirm knockdown efficiency. (**C**) Apoptosis in transfected MCF-7 cells following 24 h of EEO treatment was analyzed by Annexin V-FITC/PI staining and flow cytometry. (**D**) Apoptotic cell percentages were quantified accordingly. (**E**) Cell surface CRT expression in transfected MCF-7 cells was analyzed by flow cytometry 24 h after EEO treatment, and (**F**) the mean fluorescence intensity (MFI) was quantified. EEO-treated transfected MCF-7 cells were co-cultured with BMDCs, and (**G**) phagocytosis and (**H**,**I**) maturation of BMDCs were analyzed by flow cytometry. Black and red bars indicate siRNA-control and siRNA-CRT groups, respectively * *p* < 0.05, ** *p* < 0.01, **** *p* < 0.0001; ns, not significant, compared with the control group.

Previous research has demonstrated that CRT serves a crucial function as an “eat-me signal” during ICD processes [14,28]. To assess whether EEO-induced CRT surface exposure plays a necessary role in BMDC phagocytosis and maturation, their phagocytic activity and maturation were examined using CRT-knockdown MCF-7 target cells. As presented in Figure 4A,B, the mRNA and protein expression levels of CRT were significantly decreased compared with those in the control group. CRT knockdown did not affect EEO-induced cell death (Figure 4C,D), whereas CRT knockdown in EEO-treated MCF-7 cells substantially reduced cell surface CRT exposure (Figure 4E,F). Moreover, BMDCs exhibited significantly reduced phagocytosis of CRT-knockdown MCF-7 cells treated with EEO (Figure 4G) accompanied by reduced BMDC maturation (Figure 4H,I). Collectively, these results indicate that CRT, as an eat-me signal, plays an important role in ICD-mediated antitumor immunity by promoting phagocytosis and maturation of BMDCs.

## 4. Discussion

Despite the groundbreaking potential as a cancer treatment, cancer immunotherapy remains effective in only a limited proportion of patients with solid tumors, including breast and colon cancers, which are often characterized as immunologically “cold” tumors with low immunogenicity [4,5]. In this context, many cancer immunologists have focused on converting “cold” tumors into “hot” tumors by inducing ICD, which has emerged as a promising therapeutic strategy [29,30]. Therefore, the development of novel ICD inducers is important for enhancing antitumor immunity, given the limited number of currently available ICD-inducing agents.

EEO is a natural sterol isolated from *Gymnopilus orientispectabilis* [20], and its antitumor-related bioactivity has not yet been reported. In this study, we first demonstrated that EEO exhibits ICD-related immunostimulatory activity in breast cancer cells, representing an immunologically cold tumor. As shown in Figure 1A–D, EEO significantly reduced the viability of MCF-7 breast cancer cells, establishing its anticancer activity in this representative cold tumor model. In the MTT assay, EEO reduced MCF-7 cell viability in a concentration-dependent manner. The IC_50_ is therefore estimated to be approximately 204 µM. The working concentration of 100 µM used in this study corresponds to a sub-IC_50_ condition, which is appropriate for evaluating immunogenic cell death, since excessive cytotoxicity would result in passive release of DAMPs and confound their interpretation as ICD hallmarks. Previous studies have reported that ICD induction can involve a regulated form of cell death, such as apoptosis, necroptosis, pyroptosis, and ferroptosis [13,16]. Although these pathways collectively contribute to immunogenicity, our study focused on apoptosis because of its central role in the activity of conventional ICD inducers, such as doxorubicin [31,32], mitoxantrone [33], and oxaliplatin [34]. EEO increased the apoptotic population in MCF-7 cells by modulating key apoptosis regulators, including upregulation of the pro-apoptotic proteins p53 and Bax, downregulation of the anti-apoptotic protein Bcl-2, and cleavage of PARP, a well-established apoptosis marker [35]. Consistent with these changes, EEO treatment also significantly increased the Bax/Bcl-2 ratio, a widely used index of mitochondrial apoptotic susceptibility [36]. We also found that EEO increased the expression of the ER stress markers ATF4 and CHOP, suggesting that ER stress contributed to EEO-induced ICD. This is in line with previous reports linking ER stress-associated apoptosis to ICD induction [36]. These findings indicate that apoptosis is a major cytotoxic mechanism underlying the effects of EEO in MCF-7 cells.

Given the pro-apoptotic activity of EEO, we next investigated whether EEO exerts the canonical hallmarks of ICD in MCF-7 cells. EEO treatment markedly increased surface exposure of CRT, along with the release of extracellular ATP and HMGB1 (Figure 2). CRT plays a key role in ICD-mediated antitumor immune responses and functions as an eat-me signal for antigen-presenting cells, particularly DCs [37,38]. Surface-exposed CRT binds to its cognate receptor CD91 expressed on DCs, thereby facilitating engulfment of dying cells and subsequent activation of adaptive immunity [38]. ATP released extracellularly acts as a find-me signal through binding to P2X7R and P2Y11R receptors on DCs, promoting the chemotaxis and differentiation of immature DCs; therefore, sufficient extracellular ATP release is important for eliciting an immunogenic response [38,39]. Moreover, HMGB1 binds to TLR4 on DCs, a process that is crucial for effective antigen uptake and cross-presentation, which is essential for cell death to be perceived as immunogenic [40]. Collectively, these findings demonstrate that EEO not only induces apoptosis but also elicits the characteristic DAMP-associated events of ICD in apoptotic breast cancer cells.

Previous studies have reported that DCs play an essential role in generating efficient antitumor immune responses mediated by ICD-associated signals [41,42]. DCs recruited to the TME by ICD inducers undergo maturation and subsequently prime robust CD8^+^ CTL responses [42]. Moreover, mature DCs enhance the production of pro-inflammatory cytokines such as IL-1β and IL-6, which drive T helper 17 polarization and consequently elicit a potent inflammatory response [26,43]. To determine whether these ICD-associated signals could lead to functional immune activation, we evaluated the effects of EEO-treated MCF-7 cells on DC function. Co-culturing BMDCs with EEO-treated MCF-7 cells significantly enhanced BMDC phagocytosis (Figure 3A), consistent with the increased cell surface exposure of CRT. BMDC maturation was also observed, as evidenced by elevated expression of the classical maturation markers MHC class II and CD86 (Figure 3B–D). In addition, mature BMDCs increased the transcript levels of the proinflammatory cytokines IL-1β and IL-6 (Figure 3E,F). Collectively, these findings demonstrate that EEO-triggered tumor cell death exhibits the molecular hallmarks of ICD while also functionally reprogramming DCs into potent initiators of adaptive antitumor immunity.

Cell surface-exposed CRT on dying cells initiates their recognition and clearance by phagocytes, thereby promoting DC activation [26,28]. In addition, Kroemer et al. reported that blockade or knockdown of CRT reduced the engulfment of ICD inducer-treated cancer cells by DCs and diminished their immunogenicity [14]. To further determine the contribution of CRT to EEO-induced immune responses, we knocked down CRT expression and assessed its impact on EEO-mediated DC responses. CRT knockdown substantially reduced cell surface CRT exposure in EEO-treated MCF-7 cells and attenuated their uptake by BMDCs, accompanied by a marked impairment in DC maturation, whereas it did not affect EEO-induced cytotoxicity (Figure 4). CRT knockdown only partially inhibited phagocytosis, suggesting that other eat-me signals, such as phosphatidylserine [44], may contribute as well to the recognition and engulfment of dying cells by phagocytic cells. These results are consistent with those of previous reports [14,26] demonstrating that blockade of CRT suppresses DC-mediated phagocytosis and reduces the immunogenicity of cancer cells undergoing ICD, highlighting CRT as a critical determinant of effective DC-mediated antitumor immunity.

**Figure 5:**
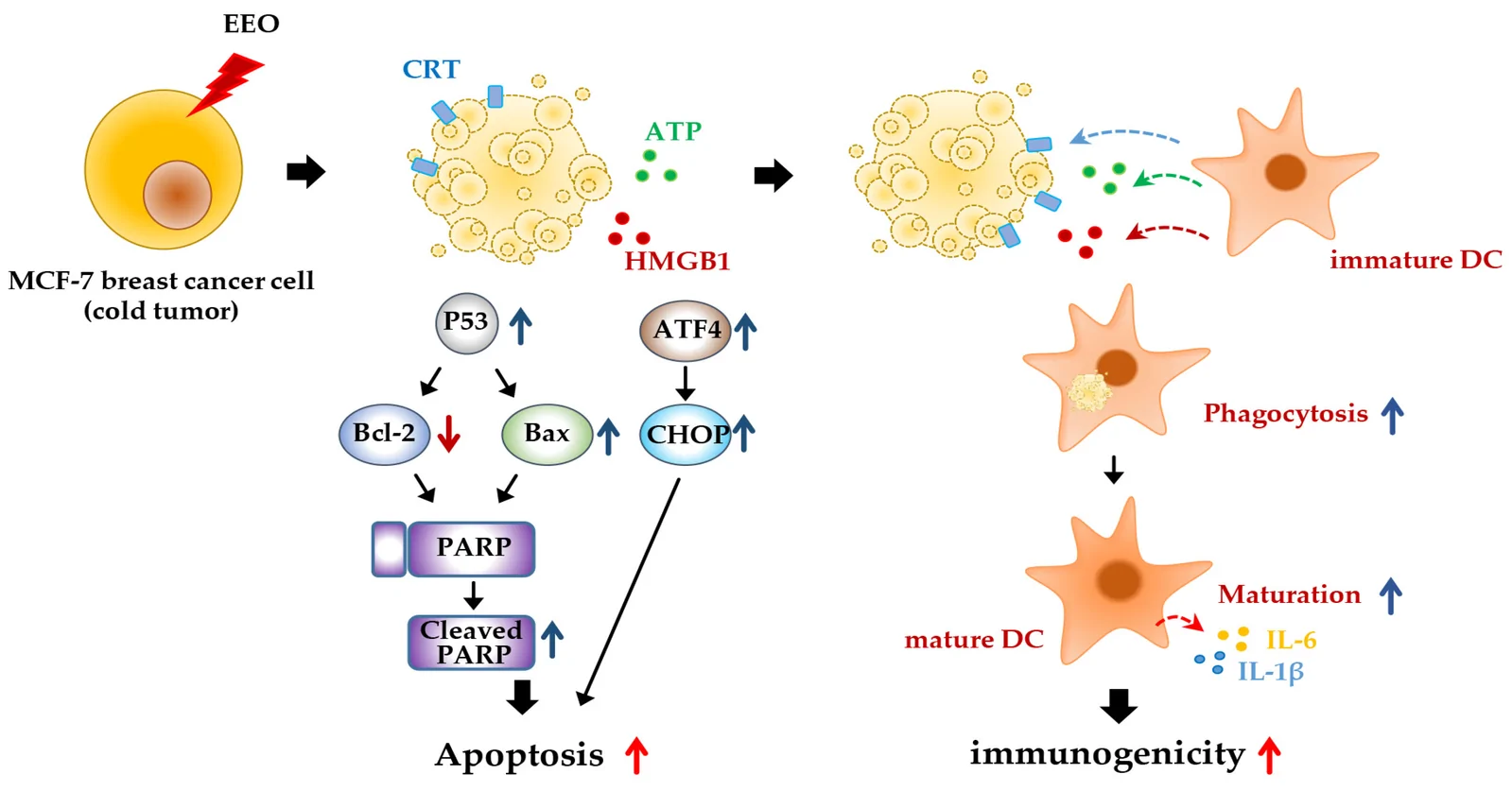
Proposed mechanism by which EEO promotes ICD and DC activation in human breast cancer cells. EEO induces apoptosis through modulation of apoptosis-related proteins (upregulation of p53, Bax, and cleaved PARP, along with the downregulation of Bcl-2) and ER stress-related proteins (upregulation of ATF4 and CHOP). EEO also promotes ICD through surface exposure of CRT and the release of DAMPs, including HMGB1 and ATP, in MCF-7 breast cancer cells. These events promote DC maturation and the engulfment of apoptotic MCF-7 cells (Abbreviations; ICD, immunogenic cell death; DC, dendritic cell; ER, endoplasmic reticulum; CRT, calreticulin; DAMPs, damage-associated molecular patterns; HMGB1, High Mobility Group Box 1; PARP, poly (ADP-ribose) polymerase; ATF4, activating transcription factor 4; CHOP, C/EBP homologous protein; Bcl-2, B-cell lymphoma 2; Bax, Bcl-2-associated X protein; IL-6, interleukin-6; IL-1β, interleukin-1 beta).

Taken together, our results demonstrate that EEO induces a CRT-dependent form of ICD in breast cancer cells, in which cell surface CRT exposure is critical for initiating an anti-tumor immune response. A schematic representation of the proposed immunostimulatory mechanism of EEO is presented in Figure 5.

## 5. Conclusions

In this study, we demonstrate that EEO induces ICD and promotes CRT-dependent DC activation, highlighting its potential as a natural ICD inducer. These findings provide a foundation for developing an EEO-based therapeutic approach aimed at converting immunologically “cold” breast tumors into “hot” tumors to enhance cancer immunotherapy. However, the therapeutic efficacy and safety of EEO-induced immunogenic cell death should be further evaluated in immunocompetent tumor-bearing animal models and clinical studies.

## Figures and Tables

**Table 1:** Sequences of primers used in qRT-PCR.

Target Gene	Primer	Sequence (5′ to 3′)
human CRT	Forward	AAGTTCTACGGTGACGAGGAG
	Reverse	GTCGATGTTCTGCTCATGTTTC
human β-actin	Forward	GGACCTGACTGACTACCTCAT
	Reverse	CGTAGCACAGCTTCTCCTTAAT
mouse IL-1β	Forward	GCCTCGTGCTGTCGGACCCATAT
	Reverse	TCCTTTGAGGCCCAAGGCCACA
mouse IL-6	Forward	TCTGAAGGACTCTGGCTTTG
	Reverse	GATGGATGCTACCAAACTGGA
mouse β-actin	Forward	ATGGAGGGGAATACAGCCC
	Reverse	TTCTTTGCAGCTCCTTCGTT

## Data Availability

The data presented in this study are available on request from the corresponding author.

## Abbreviations

The following abbreviations are used in this manuscript:

ATF4	Activating transcription factor 4
Bax	BCL2-associated X protein
Bcl-2	B-cell lymphoma 2
BM	Bone marrow
BMDC(s)	Bone marrow-derived dendritic cell(s)
BSA CD86	Bovine serum albumin Cluster of differentiation 86
CHOP	C/EBP homologous protein
CLSM	Confocal laser-scanning microscopy
CRT	Calreticulin
Ct	Threshold cycle
CTL(s)	Cytotoxic T lymphocyte(s)
DAMP(s)	Damage-associated molecular pattern(s)
DC(s)	Dendritic cell(s)
DMSO	Dimethyl sulfoxide
EEO	3β,5α,8α)-5,8-epidioxyergost-6-en-3β-ol
ER	Endoplasmic reticulum
FBS	Fetal bovine serum
GM-CSF	Granulocyte-macrophage colony-stimulating factor
HMGB1	High Mobility Group Box 1
ICD	Immunogenic cell death
IL	Interleukin
LPS	Lipopolysaccharide
MFI	Mean fluorescence intensity
MHC II	Major histocompatibility complex II
MTT	Thiazolyl blue tetrazolium bromide
PARP	Poly-(ADP-ribose) polymerase
PFA	Paraformaldehyde
PI	Propidium iodide
PSQ	Penicillin–streptomycin–glutamine
RIPA	Radioimmunoprecipitation assay
RPMI	Roswell Park Memorial Institute
RT	Room temperature
SD	Standard deviation
siRNA	Small interfering RNA
TLR4	Toll-like receptor 4
TME	Tumor microenvironment
